# A Finite Element Method for Determining the Mechanical Properties of Electrospun Nanofibrous Mats

**DOI:** 10.3390/polym16060852

**Published:** 2024-03-20

**Authors:** Jaymin Vrajlal Sanchaniya, Inga Lasenko, Valters Gobins, Alaa Kobeissi, Dmitri Goljandin

**Affiliations:** 1 Institute of Mechanics and Mechanical Engineering, Faculty of Civil and Mechanical Engineering, Riga Technical University, 6B Kipsala Street, LV-1048 Riga, Latvia; inga.lasenko@rtu.lv; 2 Laboratory of Environmental Genetics, Institute of Biology, Faculty of Biology, Latvian University, Jelgavas Street 1, LV-1004 Riga, Latvia; valters.gobins@lu.lv; 3 Université de Technologie de Compiègne, Roberval (Mechanics, Energy and Electricity), Centre de Recherche Royallieu CS 60319, 60203 Compiègne Cedex, France; alaa.kobeissi@utc.fr; 4 Department of Mechanical and Industrial Engineering, Tallinn University of Technology, Ehitajate Tee 5, 19086 Tallinn, Estonia; dmitri.goljandin@taltech.ee

**Keywords:** electrospinning, nanofibers, finite element method, oriented structures, random structures

## Abstract

This study focuses on the mechanical properties of electrospun nanofibrous mats, highlighting the importance of the characteristics of single nanofibers in determining the overall mechanical behavior of the mats. Recognizing the significant impacts of the diameter and structural properties of the nanofibers, this research introduces a novel methodology for deriving the effects of the mechanical properties of single nanofibers on the aggregate mechanical performance of electrospun oriented nanofiber mats. For this purpose, a finite element method (FEM) model is developed to simulate the elastoplastic response of the mats, incorporating the influence of structural parameters on mechanical properties. The validation of the FEM model against experimental data from electrospun polyacrylonitrile (PAN) nanofibers with different orientations demonstrates its effectiveness in capturing the elastic–plastic tensile behaviors of the material and confirms its accuracy in terms of reflecting the complex mechanical interactions within the nanofibrous mats. Through a detailed analysis of how nanofiber diameter, orientation of fibers, length-to-width ratio, and porosity affect the mechanical properties of the mats, this research provides valuable insights for the engineering of nanofibrous materials to meet specific mechanical requirements. These findings improve our understanding of nanofibrous mat structures, allowing for better performance in diverse applications as well as highlighting the critical importance of identifying the properties of single nanofibers and their associated impacts on material design.

## 1. Introduction

Electrospinning has emerged as a cost-effective and versatile methodology for the synthesis of nanofiber mats, capturing significant attention over the past two decades [1]. This technique allows for the production of nanofibrous structures through a simple single-step process in which the diameters of the fibers can be finely tuned from as small as 10 nm to several hundreds of nanometers, depending on the parameters of the process. The typical output is a nanofiber mat, although specialized collector setups can yield a variety of structures, including mats with oriented and random fibers.

Nanofibrous mats are made of fibers that are considerably longer than their diameter and appear to be virtually endless. This characteristic is a result of the electrospinning process, where a thin jet emerges from the tip of a Taylor cone, stretches, and travels towards the collector, undergoing various instabilities along the way [2].

The applications of nanofiber mats are diverse, ranging from drug delivery [3] to filtration [4,5], nanocomposites [6], wound dressings [7], energy harvesting [8], protective gear [9], and tissue scaffolding [10]. A crucial aspect of leveraging these mats in various applications is understanding their mechanical behaviors, which necessitates a deeper analysis based on the mechanical properties of the individual nanofibers.

To unlock the full potential of nanofibers, it is imperative to characterize their stress–strain behaviors at different structural levels, from the individual fibers to the mats which they form. However, due to their nanometric diameter and fragility, measuring the mechanical properties of single nanofibers is challenging. Traditional methods to determine these properties require sophisticated and expensive equipment, such as atomic force microscopes and micromanipulators [11,12,13], often limiting the scope of tests to assessing the geometric, morphological, and thermal properties. However, understanding the tensile stress of single fibers is crucial for the design of nanofibrous structures such as composites or filtration media.

The complexity and high porosity of nanofiber mats pose additional challenges in identifying the mechanical properties of individual fibers within the mats. Various researchers [14,15] have developed methods to approximate the thickness of nanofiber mats by identifying their porosity and measuring the mass of specimens in comparison to known polymer densities. When nanofibers are randomly oriented, the determination of the force acting on individual fibers is complicated; however, it becomes feasible to determine the mechanical properties of a mat when the fibers are aligned, allowing for the direct observation of the forces at play.

Finite element method (FEM) modeling has received increasing attention in research focused on analyzing the mechanical properties of fibrous non-woven mats. Studies have incorporated various assumptions and methodologies to investigate the characteristics of said mats as well as exploring the use of multi-scale modeling techniques, including statistical models, neural networks, molecular dynamics, and FEM [16,17,18,19,20,21,22,23]. Such simulations have highlighted the importance of fiber diameter, material behavior, and porosity in determining the mechanical properties of fibrous mats. Among these computational models, FEM stands out for its ability to satisfactorily describe the elastic–plastic behavior of fibrous mats by considering the mechanical behavior of embedded microelements.

Recent advances in the development of finite element (FE) models have facilitated the prediction of the mechanical properties of randomly oriented nanofibers through the examination of the mechanical properties of single nanofibers. Zhang et al. [22] have conducted a study on the mechanical response of electrospun non-woven fibrous networks under large deformations, focusing on networks composed of elastic–plastic fibers. They examined the elastic and plastic properties of these networks under uniaxial tensile loading, cyclic loading, and simple shear loading and revealed the relationship between macroscopic properties and microstructure evolution using representative volume elements (RVEs). Similarly, Yin et al. [19,20] have developed a tensile constitutive relation and an FE (finite element) model based on the testing of individual nanofibers. Their model predicted the elastic–plastic behavior of nanofiber mats, including those with fibers arranged randomly and biaxially. Chavoshnejad et al. [24] have introduced a multi-scale FE model to assess the impact of inter-fiber bonding at varying porosities on the rupture behavior of electrospun nanofiber mats. Their findings indicated that increased inter-fiber bonding enhances the stiffness and toughness of a mat, although the influence of bonding on mechanical behavior becomes less significant as the bonding percentage increases. At a certain level of porosity, a high percentage of bonding transitions the mat to an affine deformation, diminishing the effectiveness of bonding. 

A gap in the literature has been identified concerning a detailed understanding of the mechanical elastoplastic behavior of single nanofibers derived from whole nanofiber mats and the subsequent prediction of the mechanical behavior of the entire nanofiber mat from these properties. Moreover, much of the recent literature has concentrated on the random structure of nanofiber mats, often overlooking the behavior of the mat when nanofibers are oriented and inter-bonded.

In this paper, we present a novel approach that utilizes aligned nanofibers to predict the elastoplastic behavior of individual fibers. For the experimental setup, PAN nanofibers were produced, laying the groundwork for the development of the model. On the basis of these predictions, we developed an FEM model that is capable of efficiently estimating the elastoplastic behavior of nanofiber mats in the longitudinal direction. The model assesses the impacts of diameter, porosity, orientation, and length–width ratio on the mechanical properties of mats, offering a comprehensive understanding of their behavior under various conditions. 

## 2. Materials and Methods

### 2.1. Materials

The materials utilized for the fabrication of electrospun nanofibers included polyacrylonitrile (PAN) powder and N,N-dimethylformamide (DMF). Specifically, polyacrylonitrile, with an average molecular weight of 150,000 (typical) and a CAS number of 25014-41-9, along with N,N-dimethylformamide, characterized as an ACS reagent (solvent) with a purity <99.8% and a CAS number of 68-12-2, were obtained from Sigma-Aldrich Chemicals, part of Merck KGaA, located in Steinheim, Germany (postal code 89555).

### 2.2. Fabrication of PAN Nanofibers

The methodology for the manufacturing of polyacrylonitrile (PAN) nanofibers utilized an electrospinning process employing a 10% wt./wt. polymer solution, as previously described by the authors [25,26]. The electrospinning parameters were meticulously selected to optimize the fiber formation process. Specifically, a voltage of 20 kV was applied to create the necessary electric field, while a flow rate of 1 mL/h was maintained using an 18 Ga flat needle to ensure the steady extrusion of the polymer solution. The distance between the center of the syringe needle and the collector drum was set to 18 cm, a configuration which promotes the elongation and thinning of the polymer jet before deposition.

To achieve a uniform deposition, the rotating drum collector was operated at a constant speed of 2100 rpm, as mentioned in [26]. This rotation speed is critical to collecting fibers uniformly and aligning them, due to the forces exerted by the rotating surface. In addition to fabricating aligned nanofiber mats, the methodology also allowed for the production of randomly oriented nanofiber mats. For this purpose, nanofibers were collected in a flat-plate collector measuring 20 cm × 20 cm, positioned the same distance from the tip of the syringe as the drum collector.

This dual approach for the collection of nanofibers—utilizing both a rotating drum and a flat-plate collector—allows for a comparison between aligned and randomly oriented nanofiber mats. Such a comparison is invaluable for assessing the impact of fiber orientation on the mechanical properties of mats. The precise control of the electrospinning parameters and the collection methodology play a pivotal role in determining the structural characteristics of the nanofibers, which, in turn, influence their mechanical behavior. This detailed methodology ensures the reproducibility of the electrospinning process, facilitating the production of nanofiber mats of a consistent quality for further analyses of mechanical properties.

### 2.3. Morphology

The morphology of polyacrylonitrile (PAN) nanofibers was examined using scanning electron microscopy (SEM) in order to assess their diameter and orientation, which are key parameters which significantly influence their mechanical properties. For this purpose, a Hitachi TM300 tabletop SEM (Hitachi High-Tech Corporation, Tokyo, Japan) was used, operating at a magnification of 1500× and under a vacuum of 10^−2^ Torr. To prepare the samples for SEM analysis, an ion-coating process was utilized, applying a current of 6 mA to deposit a gold (Au) layer with a thickness of 150 Å on the nanofibers [6,25,26]. This coating enhanced the conductivity of the samples, thus improving the quality of the SEM images.

The fiber orientations of the nanofiber mats were quantitatively analyzed using the OrientationJ plugin within the ImageJ software suite (version 1.54h) [27,28,29], developed by the National Institutes of Health (Bethesda, MD, USA). This analysis tool allowed for a detailed assessment of the alignment of the fibers within the mats, providing information on their structural organization.

To determine the mean diameter of the nanofibers, measurements were taken from 100 randomly selected fibers across three SEM images. This approach ensured the representative sampling of the nanofiber population, allowing for the accurate calculation of the average fiber diameter and its standard deviation [25,26]. Enhancement of the contrast in the SEM images facilitated a more precise observation of the morphology of the nanofibers, enabling a detailed analysis of their structural characteristics. This comprehensive morphological assessment provided a foundational understanding of the physical properties of the fibers, which was essential for correlating their structural attributes with their mechanical behavior.

### 2.4. Porosity

A density comparison method was employed for porosity testing of the nanofiber mats, which involved measuring the mass of the nanofiber mat to determine its porosity. This method is expressed in the following equation:

(1) $$P\left(\%\right)=\left(1-\frac{M}{M_{d}}\right)\times 100,$$

where *P* represents the porosity percentage, *M* is the measured mass of the nanofiber mat (µg), and *M_d_* is the theoretical mass that the specimen would possess if it were fully dense. The value of *M_d_* is calculated using the formula *V ×* ρ, where *V* represents the volume (cm^3^) of the specimen, and ρ is its density (1.184 g/cm^3^).

The precision measurement of the mass of the nanofiber mat was facilitated through the use of a high-precision scale—specifically, the KERN ABT 5NM (KERN&Sohn GmbH, Balingen, Germany)—which features a maximum weight capacity of 100 g and a precision of 0.000001 g. The detailed calibration specifications of the scale, including its serial number (WB22G0101) and calibration certificate number (B61-389-2023-03/1, dated 24 March 2023), emphasize the meticulous approach used for these measurements. Such a precision ensured the reliability and accuracy of the data collected for the porosity analysis, providing a robust foundation for the subsequent evaluation of the properties of the nanofiber mats.

### 2.5. Tensile Test

The evaluation of the tensile properties of the polyacrylonitrile (PAN) nanofiber mats was carried out using a Mecmesin Multi-Test 2.5-i tensile testing machine, equipped with 25 N and 250 N sensors, provided by PPT Group UK Ltd., trading as Mecmesin, based in Slinfold, UK. To ensure the precision and reproducibility of the tensile tests, the samples were conditioned in an environment that complies with ISO 139:2005 [30]. This standard specifies a controlled environment with a temperature of 21 ± 1 °C, a relative humidity of the air of 60%, and an atmospheric pressure of 760 mm Hg. Such conditions are critical in minimizing the impact of environmental variables on material properties.

The dimensions of the samples prepared for tensile testing were a length of 50 mm and a width of 10 mm, according to the ASTM D882-18 standard [31]. The thickness of the nanofiber mats was precisely measured using a digital micrometer with a range of 0–25 mm (Digimatic micrometer, MDC-25PX, Mitutoyo, Kawasaki, Japan), ensuring an accurate assessment of the dimensional properties [25,26].

For a comprehensive evaluation of the mechanical behavior, the specimens were prepared in parallel and perpendicular to the nanofibers’ orientation. This preparation allowed for tensile testing in the longitudinal and transverse directions, providing insight into the anisotropic properties of the mats. Additional specimens were also prepared from randomly oriented nanofibers collected on a flat-plate collector, in order to compare the mechanical properties.

A 50 mm × 40 mm paper template, with an inside cut of 30 mm × 20 mm, was used to facilitate the handling of the specimens during the tensile test. The specimens were secured to the paper template using double-sided thin scotch tape, ensuring that both ends were firmly attached. Then, this setup was mounted onto the tensile testing machine. Before starting the test, the sides of the paper template were carefully cut with scissors, as detailed in previous studies [6,25,26], in order to ensure that the force applied during the test was transmitted directly to the specimen.

This meticulous preparation and testing protocol allowed for the precise determination of the tensile properties of the nanofiber mats, providing valuable data on their mechanical performance under various loading conditions.

### 2.6. Defining the Mechanical Properties of a Single Nanofiber

The entire process involved generating a stress–strain graph for an individual nanofiber, particularly in scenarios in which the fibers were uniformly oriented in one direction. In tensile testing, such oriented fibers carry the load uniformly. This allows for the conceptualization of the nanofiber mat as a uniform polymeric film, wherein each segment exhibits identical mechanical properties. This uniformity is presumed to hold true across the thickness of the section, down to the nanometer scale, resulting in a consistent thickness throughout.

To elucidate the mechanical properties of individual nanofibers within the oriented nanofiber mats, a detailed methodological approach was adopted, focusing on the characterization of the mat’s mechanical behavior as a proxy for the properties of a single fiber. Following the tensile testing of the mats, the mass of each specimen was meticulously measured, adopting the premise that the density of the specimens is equivalent to that of pure polyacrylonitrile (PAN). Given the known dimensions of the tested specimens—specifically, a length of 30 mm between the grips and a width of 10 mm—a re-calculated thickness was derived based on the density of PAN. This re-calculated thickness facilitated the plotting of stress–strain graphs for the individual nanofibers, taking into account the elastoplastic behavior of each nanofiber. For the precise measurement of each specimen’s weight, a laboratory scale with a high degree of accuracy was used (as mentioned in Section 2.4).

## 3. Finite Element Model

### 3.1. Geometric Modeling

**Figure 1:**
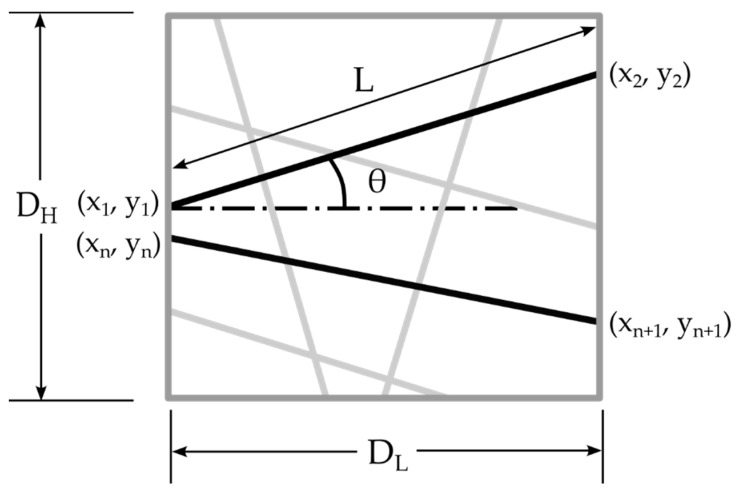
Schematic representation of the geometry development process for nanofibers, highlighting the control of fiber orientation and distribution within the domain.

For the development of the FE model, a detailed geometric modeling approach was employed, focusing on the creation of a parametric model to accurately represent the structure of the nanofibers. Figure 1 illustrates the controlled parameters, which are essential for the geometric development of nanofibers, thus laying the foundation for the FE model. The process of generating this parametric model began with the construction of straight fibers possessing a uniform diameter, which were evenly distributed across a defined plane. This distribution was characterized according to the known length of the domain (D_L_) and the height of the domain (D_H_).

The methodology for developing this geometry involves several key steps:

- All fibers are initially generated with their midpoint located at the center of the domain area.
- Each fiber is then rotated at a random angle ranging from −90 to +90 degrees, with the fiber length extending to the boundaries of the domain.
- For oriented nanofibers, the angle of fiber rotation is precisely controlled to reflect their aligned structure.

Control of the porosity within the domain was achieved through controlling the domain area and obtaining the coordinates of the generated fibers. As depicted in Figure 1, for a fiber generated at an angle (θ), its starting coordinates are indicated as (x_1_, y_1_) and its ending coordinates are (x_2_, y_2_). Using the angle (θ) and these known coordinates, it is possible to calculate the length (L) of the fiber within the domain area (D_L_ × D_H_). Given that the fiber diameter is also known, this methodology allows for the precise maintenance of the domain’s porosity.

This geometric model is based on four critical assumptions:

- All fibers extend to the end of the domain.
- All fibers have an equal diameter.
- All fibers maintain a straight configuration throughout the domain, without any bending.
- All intersecting fibers are inter-bonded within the domain area.

The assumption that the interlocking fibers are inter-bonded is grounded in previous research findings [24,32], indicating that a high percentage of inter-bonded fibers gives a more accurate result regarding the mechanical properties of nanofiber mats. On the basis of this observation, it was assumed that all the fibers were inter-bonded. Given the focus of this research on loading in the transverse direction and the analysis of the mechanical properties under such conditions, it was crucial to model the fibers as being inter-bonded. This assumption played a significant role in the accurate representation of the nanofiber mat’s behavior, especially under transverse loading conditions, where the inter-bonding between fibers could influence the distribution of stress and strain throughout the material.

To facilitate this geometric development process, an auxiliary Python code was developed, as mentioned in [22,24], which enabled the efficient and accurate construction of the model’s geometry. This approach ensured that the FE model accurately reflected the physical characteristics of the nanofiber mat, providing a robust basis for the subsequent mechanical analyses.

### 3.2. Boundary Conditions

**Figure 2:**
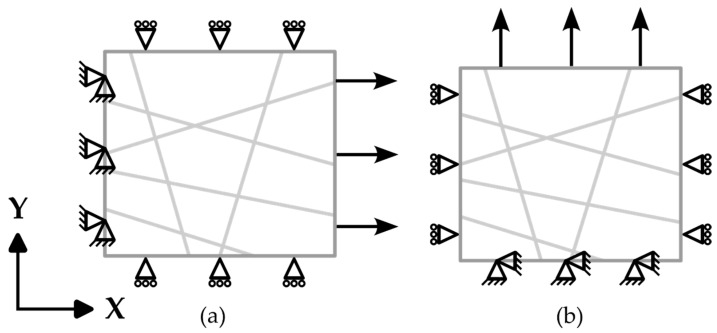
Boundary conditions applied in (**a**) longitudinal and (**b**) transverse directions to simulate the normal stress response to displacement.

In the methodology for analyzing the mechanical behavior of nanofiber mats under tensile load, specific boundary conditions were implemented to simulate the response to longitudinal and transverse displacements, as depicted in Figure 2. These conditions were designed to observe the reaction force (RF) resulting from the displacement applied in the longitudinal direction. When displacement was applied to the ends of the fibers, it extended up to 20% of the domain’s length, with constraints applied to prevent movement in the Y and Z directions (U2 = U3 = 0) for longitudinal loading (Figure 2a) and in the X and Z directions (U1 = U3 = 0) for transverse loading (Figure 2b). The fiber ends opposite the direction of displacement were fixed in all directions (U1 = U2 = U3 = 0) but were allowed rotational freedom around the *Z*-axis, accounting for the change in angle relative to the *Z*-axis as displacement occurred. The edges perpendicular to the displacement direction were allowed to move freely in alignment with the displacement, while being restricted from sliding towards each other. For displacement along the *X*-axis, the top and bottom boundaries were set with the conditions U2 = U3 = 0; similarly, for displacement along the *Y*-axis, the left and right edges satisfied the conditions U1 = U3 = 0.

For the finite element analysis, the fibers were modeled using linear beam elements (B31) within the Abaqus finite element software package (2022). These linear beams are shear-deformable and accommodate finite axial strains, making them suitable for modeling slender structures such as nanofiber mats.

After applying the boundary conditions, the reaction force of each fiber was observed with respect to the X-axis, and the sum of these reaction forces was divided by the domain’s cross-sectional area, as shown in Equation (2) below, in order to plot the elastoplastic behavior of the nanofiber mat. Similarly, when the fibers were randomly structured, reaction forces were observed in both the *X*- and *Y*-axes. This method allowed for the direct measurement of the stress within the mat, facilitating a detailed analysis of its mechanical response under tensile loading. Through aggregating the reaction forces and normalizing them over the cross-sectional area, a comprehensive understanding of the mat’s stress distribution and its elastoplastic behavior was achieved, providing valuable insights into the material properties of the nanofiber mat. The following relationship formed the basis for calculating the stress within the nanofiber mat, providing a clear and quantifiable measure of the material’s response to applied displacement.

(2) $$\sigma_{x}=\frac{\sum F_{x}}{A_{D}},$$

where $\sigma_{x}$ is the normal stress in *X*-axis, $F_{x}$ is the reaction force (µN) acting at each end of the fibers, and $A_{D}$ is the cross-sectional area of the domain. 

Given the inherent variability when generating fibers with specified combinations of orientation and porosity, multiple structures can result from each set of parameters. This variability introduces a degree of uncertainty in the mechanical properties derived from a single simulation. To mitigate this problem and ensure the reliability and robustness of the mechanical properties obtained, at least five simulations were performed for each combination of parameters. This approach allowed for a comprehensive analysis that encompassed the range of possible structures, ensuring that the derived mechanical properties accurately reflected the behavior of the nanofiber mats under different conditions. Through averaging the results of these simulations, this study provided a more robust and reliable understanding of the mechanical behavior of the mat, accounting for the variations in the structure which occurred due to differences in fiber orientation and porosity.

### 3.3. Material Model

The values used in the FEM results section were derived from the mechanical properties of individual nanofibers, providing a foundational understanding of the material’s behavior under stress on the basis of three key parts: namely, addressing the elastic behavior, plastic behavior, and damage model of the nanofiber mat material. The elastic behavior of the material is described in Equation (3), which relates stress (σ) in the material to strain (ε) through Young’s modulus (E), where ε_y_ is the yield strain:

(3) $$\sigma =E\;\cdot \;\varepsilon \;for\;\varepsilon \leq \;\varepsilon_{y}$$

The non-linear behavior of the elastic–plastic transition is described in Equation (4):

(4) $$\varepsilon =\frac{\sigma }{E}+{\left(\frac{\sigma }{K}\right)}^{n},$$

where *K* is the strength coefficient, and *n* is the hardening exponent. 

For the damage model, which predicts the energy which must be absorbed by the individual nanofiber to fail under stress, Equation (5) is used, which is based on the concept of fracture energy and linear plastic displacement:

(5) $$G_{f}=\frac{1}{2}\sigma_{f}\cdot \delta_{f},$$

where *G_f_* is the fracture energy (µN/µm) representing the energy absorbed by the individual nanofiber until failure, *σ_f_* is the stress at failure, and *δ_f_* is the linear plastic displacement at failure. 

Together, these equations formed the basis for the material model used in this study, enabling a comprehensive analysis of the mechanical behavior of the nanofiber mats from elastic response to plastic deformation and ultimate failure.

## 4. Results and Discussion 

### 4.1. Morphology

**Figure 3:**
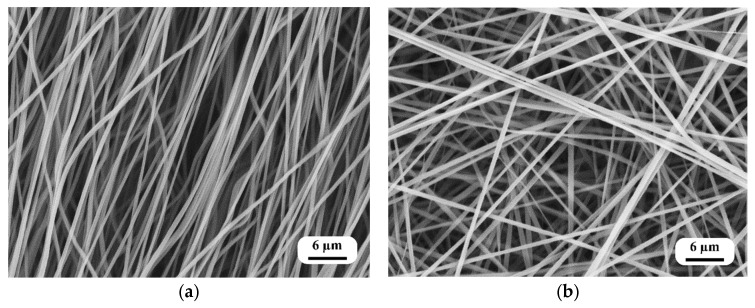
SEM images of the nanofiber mats: (**a**) oriented nanofibers and (**b**) random nanofibers.

Figure 3a,b show the SEM images of the nanofiber mats, which highlight the distinct differences between the oriented and randomly structured nanofiber mats. The diameter of the nanofibers in the oriented structure was found to be 580 ± 20 nm, while the diameter in the random structure was slightly larger, 719 ± 35 nm. This variation in diameter could be attributed to the collection methods employed during the electrospinning process. Specifically, the nanofibers collected from the rotating drum—similar to those previously studied by the authors [25]—maintained a narrower diameter due to the drawing effect induced by the high-speed rotation of the drum. In contrast, the nanofibers collected using the flat-plate collector exhibited a larger diameter, likely due to the absence of the drawing effect present in the rotating drum collection method.

**Figure 4:**
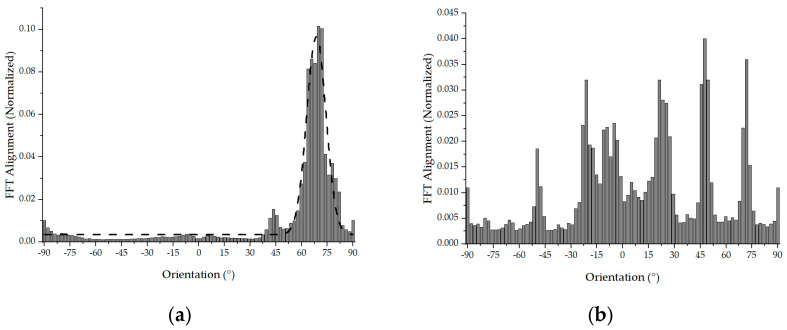
Orientation of the nanofibers: (**a**) oriented nanofibers and (**b**) random nanofibers.

Figure 4a,b show the results of the fast Fourier transform (FFT) analysis, which was used to quantify the alignment of the nanofibers within the mats. For the oriented nanofiber mats collected at a drum speed of 2100 rpm, the FFT alignment value (normalized) ranged from 0.0 to 0.1, indicating a high degree of alignment, with most nanofibers oriented in a single direction and varying within a range of 11 degrees. This consistent alignment was facilitated by the constant rotational speed of the drum, which stabilized the fiber-drawing process and resulted in a uniform diameter range. On the contrary, in the randomly structured mats, the FFT alignment value (normalized) exhibited a smaller fluctuation, from 0.01 to 0.04, suggesting that the fibers were distributed more randomly throughout the mat and did not exhibit a strong directional preference.

Using the density comparison method (outlined in Equation (1)), the porosity of the oriented nanofibers was determined to be 75.2 ± 1%, while the porosity of the random nanofiber mats was slightly higher, at 80.8 ± 1%. The increased porosity observed in the flat-plate collector samples could be explained by the lower packing density of the nanofibers, compared to those collected on the rotating drum, which were more tightly drawn and aligned.

### 4.2. Experimental Results of the Mechanical Properties of a Nanofiber Mat

**Figure 5:**
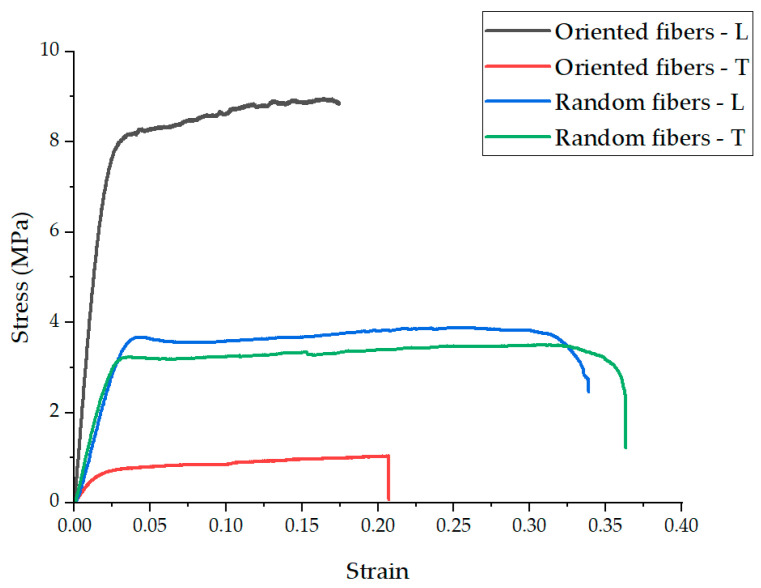
Representative stress–strain graphs for oriented and random structures in the longitudinal (L) and transverse (T) directions.

Figure 5 presents the stress–strain curves for specimens with oriented nanofiber structures, illustrating the material’s response under tensile loading. The ultimate tensile strength (UTS) of the oriented nanofibers in the direction of the fibers was recorded to be 8.9 ± 0.5 MPa, demonstrating a significant strength advantage over the transverse direction, where the UTS was much lower, at 1.1 ± 0.1 MPa. This directional discrepancy in strength highlighted the anisotropic nature of the oriented nanofiber mats. On the contrary, for the randomly structured nanofiber mats, the UTS was relatively uniform in both directions, with values of 3.9 ± 0.4 MPa and 4.0 ± 0.5 MPa, respectively. This near-equivalence in strength regardless of the direction highlighted the isotropic mechanical behavior of the randomly structured mats.

Young’s modulus (a measure of stiffness) for the oriented nanofiber mats was significantly higher in the longitudinal direction (410 ± 23 MPa) compared to the transverse direction (53 ± 5 MPa), further emphasizing their anisotropic characteristics. For the random nanofiber mats, Young’s modulus was more consistent in both directions, with values of 103 ± 4 MPa and 99 ± 5 MPa, indicating a more uniform distribution of stiffness throughout the material.

The elongation at break (an indicator of ductility) varied between the structures and directions. The oriented nanofibers exhibited an elongation at break of 0.19 ± 0.02 in the longitudinal direction and 0.2 ± 0.03 in the transverse direction. Randomly structured nanofibers showed a higher ductility, with elongation at break values of 0.35 ± 0.03 and 0.36 ± 0.04.

The increased plasticity observed in the random structures (i.e., the nanofiber mat) could be attributed to the inter-bonding of fibers at various angles, contributing to greater elongation before failure. In a random structure, not all fibers are aligned or subjected to load in the same manner, which is expected to result in a lower elastic modulus and a lower ultimate tensile strength compared to oriented structures. On the contrary, in the case in which the fibers are oriented, they all carry the load and break together. This collective load-bearing behavior of oriented fibers contributes to their higher mechanical strength and stiffness in the direction of their alignment, underscoring the anisotropic mechanical properties which are characteristic of these oriented nanofiber mats. Table 1 summarizes the mechanical properties of all nanofiber mats.

The results of this test revealed the significant impact of fiber orientation on the mechanical properties of nanofiber mats. The oriented structures demonstrated superior mechanical properties in the direction of fiber alignment, indicative of their potential for applications requiring high tensile strength and stiffness in a specific direction. In contrast, the randomly structured mats offered more balanced properties, making them suitable for applications where isotropic mechanical behavior is desired.

### 4.3. Predicted Stress–Strain Curve of Single Nanofibers

The investigation of the mechanical properties of nanofiber mats further extended to an analysis of the predicted stress–strain behavior of single nanofibers, particularly after addressing the effect of porosity within the mats.

**Figure 6:**
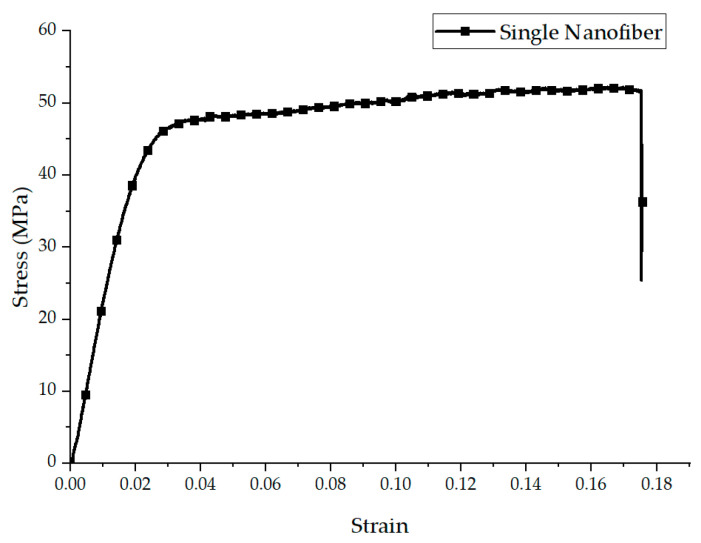
Representative stress–strain graph for a single nanofiber.

Figure 6 shows the modeled elastoplastic behavior of a single nanofiber, derived through theoretically eliminating the influence of porosity from the nanofiber mats. This approach allowed for a more direct assessment of the intrinsic mechanical properties of the nanofibers, independent of the structural characteristics of the mats.

The analysis revealed that the elastic modulus of a single nanofiber was significantly higher than that observed in the bulk mats, with a value of 2100 ± 110 MPa. This substantial increase highlights the inherent strength and stiffness of individual nanofibers, which may be diluted in bulk materials due to the presence of porosity and the distribution of fibers. Furthermore, the ultimate tensile strength (UTS) of the single nanofiber was determined to be 51.9 ± 5 MPa. This value, derived from the data on the elongation at break of the oriented nanofibers, reflects the robust tensile capacity of the fibers, with the elongation at break strain remaining constant at 0.19 ± 0.02.

The mechanical properties of a single nanofiber were used to validate the finite element (FE) model and predict the mechanical behavior of the nanofiber mat. This approach ensured that the theoretical and computational models accurately reflected the intrinsic properties of the nanofibers, allowing for a more precise simulation of the response of the nanofiber mats under different loading conditions. With the properties of the single fibers being compared to the overall behavior of the mats, this method provided a robust framework for understanding and predicting the performance of nanofiber-based materials in practical applications.

### 4.4. Convergence and Validation of the Developed FEM Model

**Figure 7:**
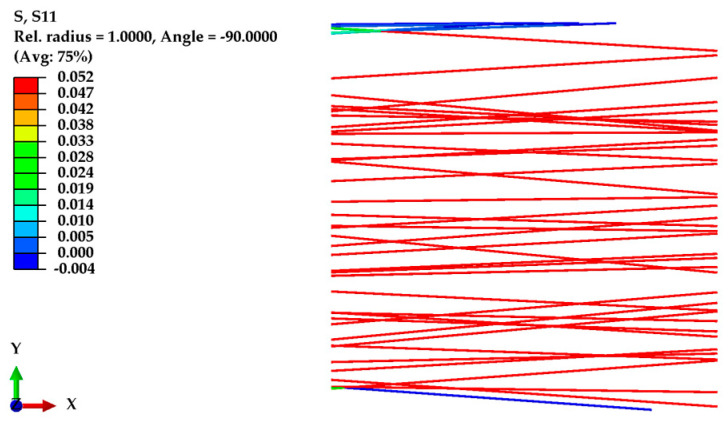
Normal stress along the X-axis for an oriented nanofiber mat under displacement.

To validate the finite element (FE) model, the initial model was developed based on experimentally obtained data. This model had a domain size of 100 μm × 100 μm, incorporating a 75% porosity level. All the fibers were aligned within a narrow angular range of 11 degrees (from +5.5 degrees to −5.5 degrees), each having an equal diameter of 580 nm and a fiber length between 70 μm and 100.47 μm (depending on the angle (θ) with the X-axis). The elastic modulus of the nanofibers was considered to be 2100 MPa, and their Poisson’s ratio was assumed to be 0.4, providing essential material properties for the simulations. A displacement of 20 μm (20% of the domain length) was applied along the X-axis, with the fracture energy required for breakage set to 13 µN/μm. For simplicity, all the units in the Abaqus simulations were maintained in micrometers (μm). Figure 7 shows the normal stress in the X-axis for an oriented nanofiber mat under displacement.

**Figure 8:**
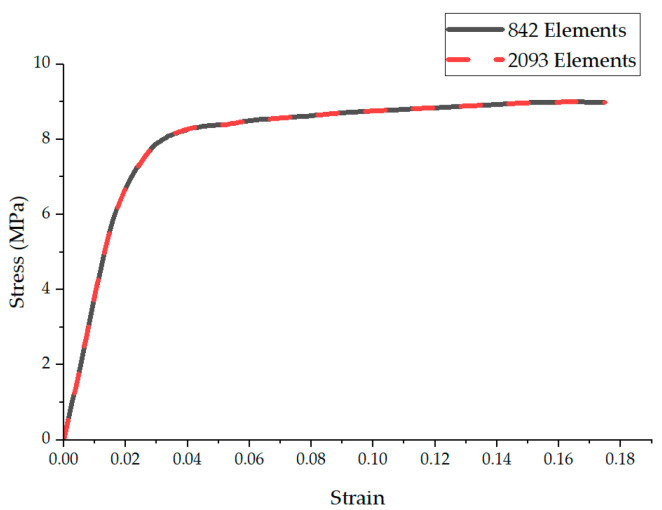
Mesh refinement of the FE model for oriented nanofibers.

Figure 8 illustrates the effect of mesh refinement on the developed FE model, using the same geometry and parameters but with approximately 2.5 times more elements. The graph compares the normal stress–strain response for systems with 842 elements (represented by a black line) and 2093 elements (indicated by a red dashed line). Interestingly, the graph demonstrates that an increase in the number of elements did not significantly impact the stress–strain behavior of the oriented fibers, which could be attributed to the reaction force being calculated relative to the domain’s cross-sectional area.

**Figure 9:**
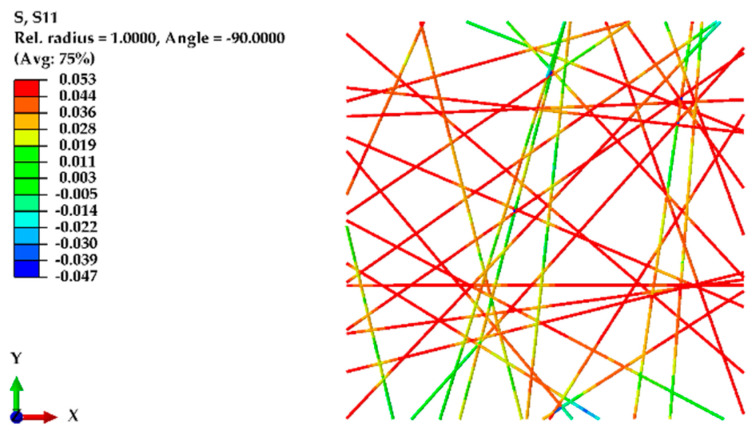
Normal stress along the *X*-axis in a random structure of nanofibers under *X*-axis displacement.

**Figure 10:**
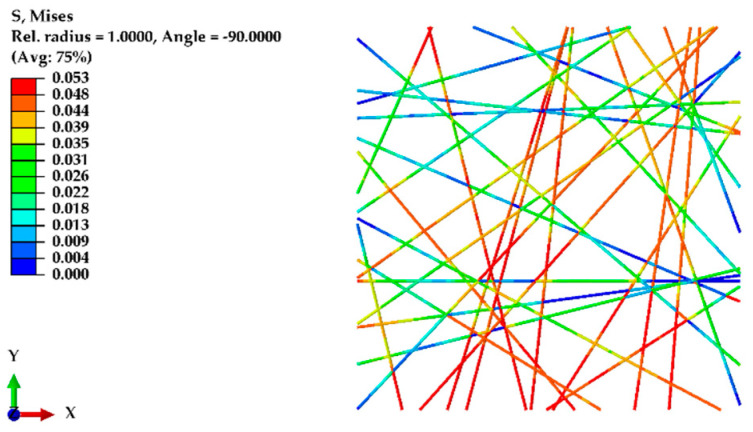
von Mises stress in random fibers under *Y*-axis displacement.

To model the random structure of nanofibers and validate the experimental results, a similar domain size of 100 μm × 100 μm was used, this time considering an 80% porosity, fiber diameters of 720 nm, and a fiber length between 30 μm and 141.44 μm (depending on the angle (θ) with the X-axis and Y-axis). The fibers were randomly distributed within the domain. Displacements were applied along the X-axis and, for comparison, along the Y-axis as well. Figure 9 shows the normal stress along the X-axis in the random structure of nanofibers, while Figure 10 shows the von Mises stress in the random structure of nanofibers under displacement along the Y-axis.

**Figure 11:**
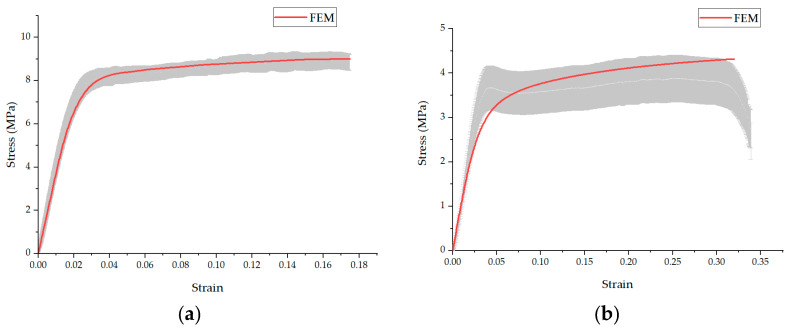
Comparison of FE model predictions for (**a**) oriented and (**b**) randomly structured nanofiber mats, highlighting its accuracy in simulating mechanical behaviors. Grey region represents the experimental results with the standard deviation.

Figure 11 compares the predictions of the FE model regarding the behavior of nanofiber mats with oriented and randomly structured nanofibers. In the case of the random structure, the experimental results indicated similar mechanical responses in both the transverse and longitudinal directions. The FE model accurately predicted this behavior, showing nearly identical stress responses along both the X- and Y-axes for the random structure.

For the transverse direction, the strength of oriented fibers usually depends on the strength required to break the bonds between the fibers as there are no fibers along the transverse direction, resulting in no reaction forces in the transverse direction. This method is limited to predicting the strength of the fibers in which at least a few fibers are in the loading direction, highlighting a critical aspect of the model’s applicability in assessing the mechanical properties of nanofiber mats with varied orientations.

In the analysis of random nanofiber mats, the experimental results revealed a peak in the elastic zone, which could be attributed to the presence of a few aligned fibers within the system. Indeed, these fibers break upon reaching their tensile strength limit, transferring the load to neighboring fibers oriented at various angles. Within the framework of the finite element (FE) model, once they reach their failure point, these initially aligned fibers are considered to be damaged and cease to contribute to the load-bearing function. Subsequently, the load is re-distributed to the angular fibers, which then carry the load further.

This behavior is precisely why, in the finite element (FE) model, a smooth transition from the elastic zone to the plastic zone is observed, mirroring the nuanced mechanical response of nanofiber mats under a load. Unlike the experimental results, where a distinct peak was evident in the stress–strain curve (attributable to the initial failure of aligned fibers), the FE model delineates a more gradual shift from elastic deformation to plastic flow. The obtained results are in agreement with those previously obtained in various studies [20,22,24,32,33]. This discrepancy arises from the model’s capacity to simulate the progressive damage and failure of individual fibers as well as the subsequent re-distribution of stress among the remaining intact fibers, which may not be as easily discernible in physical experiments.

### 4.5. Effect of Structural Parameters on the Strength of the Nanofiber Mat

To investigate the impact of structural parameters on the elastic modulus and ultimate tensile strength (UTS) of nanofiber mats, a systematic study was conducted by varying one parameter at a time and keeping the others constant. The domain size was set to 100 µm × 100 µm, with material properties derived from the experimental results for single nanofibers. For the oriented fibers, the alignment was within 10 degrees, while the random fibers were generated without restrictions. The porosity of the mat was fixed at 75%, and the general diameter of the nanofibers was considered to be 600 nm.

**Figure 12:**
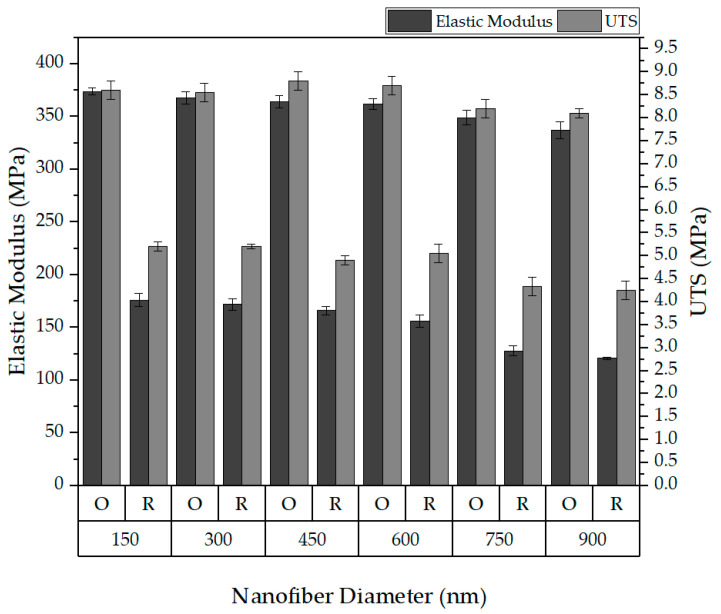
Effect of the nanofiber diameter on elastic modulus and UTS.

The effect of the nanofibers’ diameter was examined in the range from 150 nm to 900 nm for both the oriented (O) and random (R) structures. Figure 12 reveals that, for the oriented nanofibers with a diameter of 150 nm, an elastic modulus of 374 ± 3 MPa and a UTS of 8.6 ± 0.2 MPa were obtained. In contrast, for the nanofibers with a diameter of 900 nm, the elastic modulus slightly decreased to 337 ± 7.8 MPa, with a UTS of 8.1 ± 0.1 MPa. This decrease in the mechanical properties with an increasing diameter can be attributed to the tendency of fibers with a larger diameter to touch the top or bottom edges of the domain, effectively removing them from the load-bearing structure—a phenomenon which is less impactful for smaller-diameter fibers, as very few fibers reach the top and bottom edges.

**Figure 13:**
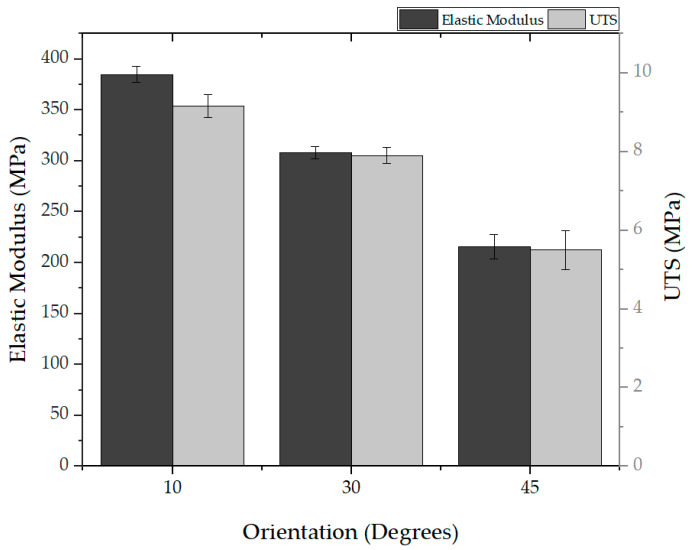
Effect of orientation on the elasticity and UTS.

The influence of fiber orientation was also explored, considering orientations of 10 degrees, 30 degrees, and 45 degrees. Figure 13 shows that fibers within a 10-degree range exhibited an elastic modulus of 385 ± 8 MPa and a UTS of 9.16 ± 0.3 MPa, while, in a 45-degree range, the elastic modulus was significantly lower (216 ± 12 MPa, with a UTS of 5.5 ± 0.5 MPa), indicating the critical role of fiber orientation in the mechanical properties of the mat.

**Figure 14:**
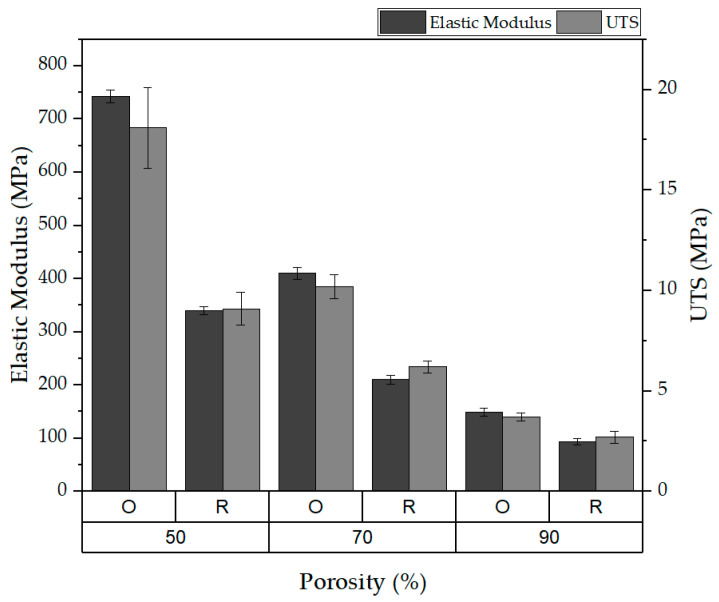
Effect of porosity on elasticity and UTS.

We also examined the effects of 50%, 70%, and 90% porosity levels on the mechanical properties. Figure 14 illustrates that, at a 50% porosity, the elastic modulus and UTS for the oriented fibers were markedly higher (743 ± 12 MPa and 18.1 ± 2 MPa, respectively) than those for the random structure (340 ± 8 MPa and 9.1 ± 0.8 MPa, respectively), demonstrating that increased porosity leads to a reduction in mechanical properties for both fiber structures. 

For the porosity, the results of this study aligned with our intuitive expectations: mats with a lower porosity, characterized by a higher density of fibers, demonstrate greater stiffness and strength compared to their more porous counterparts. A notable trend in our study was the fairly linear decrease in mat stiffness as porosity increased, underscoring the direct impact of fiber density on the mechanical properties of the mats [22].

Finally, the impact of varying the length-to-width (L:W) ratio was investigated by adjusting the domain length between 200 µm and 300 µm while maintaining a constant height.

**Figure 15:**
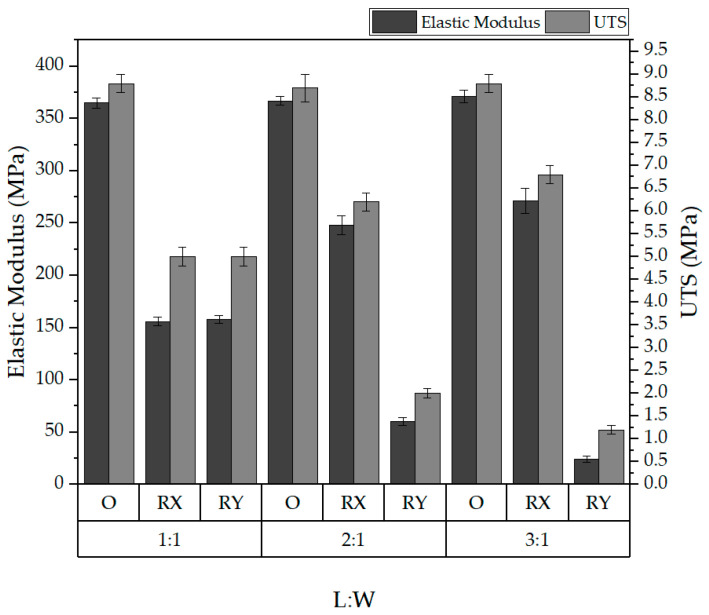
Effect of the L:W ratio on elastic modulus and UTS.

Figure 15 shows that changes in the L:W ratio significantly affected the mechanical properties of the random structure, indicating anisotropic behaviors as the ratio varied. However, the oriented fibers maintained consistent results, highlighting the stability of their mechanical properties despite changes in the L:W ratio.

Regarding the length-to-width (L:W) ratio, the findings indicated a decrease in the elastic modulus with an increase in the L:W ratio for the random structures. This observation suggests that, as the proportionate length of the domain increases relative to its width, the overall structural integrity diminishes in terms of elasticity. This effect can be attributed to the increased likelihood of a non-uniform stress distribution and the potential for localized deformation in elongated structures.

This comprehensive analysis demonstrated how the structural characteristics of nanofiber mats, such as diameter, orientation, porosity, and L:W ratio, significantly influence their mechanical properties. Randomly structured nanofibers exhibit anisotropic behaviors with varying L:W ratios, whereas oriented fibers show consistent mechanical properties, highlighting the importance of structural design in developing nanofiber-based materials for specific applications.

This study, while comprehensive, was subject to several limitations inherent to computational modeling approaches. A key assumption in the model was a perfect bonding condition of 100%, which neglects the possibility of de-bonding among intersecting fibers. In practical scenarios, the absence of an adequate bonding strength can lead to bond failures and fractures, significantly influencing the mechanical behavior of nanofiber mats [34]. The assumption of perfect bonding used in this study does not account for the variability in bond strength and its potential impact on the structural integrity of the mats.

Furthermore, the model neglected the friction between unbonded and contacting fibers. In reality, there exists slippage between these fibers, which can significantly affect the load distribution and mechanical response of mats under stress [35,36]. The absence of frictional considerations in the model simplifies the interaction between fibers but fails to capture the complex dynamics of fiber movement and interaction under loads.

Another limitation is that the model exclusively utilized straight fibers, disregarding the influence of fiber curliness or curvature on the mechanical properties of the mats. Previous research has demonstrated that the geometric characteristics of fibers, including their curvature, play a critical role in determining the overall mechanical performance of fiber mats [16,37,38]. The omission of fiber curliness from the model may, therefore, limit the accuracy of the predicted mechanical behavior.

## 5. Conclusions

In this study, the mechanical properties of electrospun nanofiber mats were comprehensively investigated using a finite element method (FEM) to elucidate the influence of structural parameters, such as fiber diameter, orientation, porosity, and length-to-width (L:W) ratio, on the elastic modulus and ultimate tensile strength (UTS) of the mats. Through experimental validation and predictive modeling, this study offers significant insights into the behavior of oriented and randomly structured nanofiber mats under various loading conditions.

The findings demonstrate that the mechanical properties of nanofiber mats are significantly influenced by their structural characteristics. The oriented nanofibers exhibited superior mechanical strength and stiffness in the direction of their alignment, as evidenced by their higher elastic modulus and UTS values, compared to the randomly structured mats. This anisotropic behavior indicates the potential of oriented nanofiber mats for use in applications requiring directional strength and stiffness.

In contrast, the randomly structured nanofiber mats showed a more isotropic mechanical behavior (at a 1:1 scale), with slight variations in their mechanical properties when subjected to changes in their structural parameters. The experimental results revealed a peak in the elastic zone for the random mats, attributed to the initial failure of the aligned fibers, which then transferred the load to fibers at various angles. This behavior highlighted the complex load distribution and resilience of nanofiber mats as a result of inter-bonding and diversity in the orientation of the fibers.

This study also explored the effect of the L:W ratio on the mechanical properties of nanofiber mats, revealing significant changes in the mechanical behavior of the randomly structured mats with varying domain dimensions. This observation points to the anisotropic behavior of random mats under different L:W ratios, contrasting the consistent performance of oriented fibers regardless of the domain shape.

This research provided a detailed analysis of the mechanical properties of electrospun nanofiber mats, highlighting the pivotal roles of certain structural parameters in determining the mechanical behavior of the mats. The validated FE model offers a valuable tool for predicting the performance of nanofiber mats, allowing for the design and development of non-woven materials for specific applications.

## Figures and Tables

**Table 1:** Summary of the mechanical properties of nanofiber mats.

Structure of Nanofibers	Testing Direction	Thickness, t (µm)	Ultimate Tensile Strength σ_max_ (MPa)	Young’s Modulus, E (MPa)	Elongation at Break Strain, ε
oriented	longitudinal	84 ± 4	8.9 ± 0.5	410 ± 23	0.19 ± 0.02
	transverse	83 ± 3	1.1 ± 0.1	53 ± 5	0.2 ± 0.03
random	longitudinal	87 ± 5	3.9 ± 0.4	103 ± 4	0.35 ± 0.03
	transverse	85 ± 4	4.0 ± 0.5	99 ± 5	0.36 ± 0.04

## Data Availability

Data are contained within the article.
